# Patients’ Views on the Design of DiabeText, a New mHealth Intervention to Improve Adherence to Oral Antidiabetes Medication in Spain: A Qualitative Study

**DOI:** 10.3390/ijerph19031902

**Published:** 2022-02-08

**Authors:** Rocío Zamanillo-Campos, Maria Jesús Serrano-Ripoll, Joana Maria Taltavull-Aparicio, Elena Gervilla-García, Joana Ripoll, Maria Antonia Fiol-deRoque, Anne-Marie Boylan, Ignacio Ricci-Cabello

**Affiliations:** 1 Research Group in Primary Care and Promotion—Balearic Islands Community (GRAPP-caIB), Health Research Institute of the Balearic Islands (IdISBa), 07120 Palma de Mallorca, Spain; mariajesus.serranoripoll@ibsalut.es (M.J.S.-R.); joanamaria.taltavull@ibsalut.es (J.M.T.-A.); joana.ripoll@ibsalut.es (J.R.); ignacio.ricci@ibsalut.es (I.R.-C.); 2 Primary Care Research Unit of Mallorca (IB-Salut), Balearic Health Service, 07002 Palma de Mallorca, Spain; 3 Primary Care Preventive and Health Promotion Research Network (redIAPP), 08007 Barcelona, Spain; 4 Psychology Department, University of the Balearic Islands (UIB), 07120 Palma de Mallorca, Spain; elena.gervilla@uib.es; 5 Statistical and Psychometric Procedures Applied in Health Science, Health Research Institute of the Balearic Islands (IdISBa), 07120 Palma de Mallorca, Spain; 6 Nuffield Department of Primary Care Health Sciences, University of Oxford, Oxford OX2 6GG, UK; anne-marie.boylan@phc.ox.ac.uk; 7 Biomedical Research Center in Epidemiology and Public Health (CIBERESP), 28029 Madrid, Spain

**Keywords:** type 2 diabetes, SMS, self-care, mobile health, eHealth, qualitative research

## Abstract

Background: Type 2 Diabetes Mellitus (T2DM) is a long-term condition affecting around 10% of people worldwide. This study aimed to explore T2DM patients’ views on DiabeText, a new text messaging intervention to be developed to support adherence to diabetes medication. Methods: A total of four focus groups were conducted with a purposive sample of people with T2DM (n = 34). The data were analysed by multiple researchers independently, and coded using thematic analysis. Results: There were two main themes that emerged: (1) “patients’ perspectives on unmet needs for diabetes self-management”, and (2) “acceptability and perceived utility of DiabeText”. The patients identified a number of barriers for diabetes self-management, including lack of appropriate information and support with diet and physical activity. Support for medication-taking was not perceived as urgently needed, although several barriers were identified (eating outside, traveling, polymedication, dispensation at the pharmacy). The participants anticipated that the proposed intervention would present high levels of patient acceptability and perceived utility as long as its content addresses the barriers that were identified, and includes specific features (short and clear messages, and personalized information). Conclusion: The proposed intervention has the potential to be well accepted and perceived as useful by T2DM patients who require support not only in terms of medication-taking, but more prominently of lifestyle behaviour.

## 1. Introduction

According to recent figures from the International Diabetes Federation [1], the worldwide prevalence of type 2 diabetes mellitus (T2DM) in 2021 was around 10%, affecting 537 million adults. This number is predicted to rise to 643 million by 2030 and 784 million by 2045. T2DM is a major cause of blindness, kidney failure, heart attacks, stroke, and lower limb amputation. As the prevalence of T2DM increases globally, health services in most countries are struggling with the morbidity, mortality, and costs that are associated with this condition [2]. Alongside lifestyle changes, medicines are used to lower blood glucose, blood pressure, and lipids to prevent long-term complications. However, international studies show that up to 37% of patients with T2DM stop their blood glucose-lowering medicine within one year of starting treatment [3], and adherence falls further as the number of tablets increases [4]. In Spain (with a prevalence of T2DM around 15%) [1], the non-adherence rates to oral antidiabetic drugs are particularly high, ranging from 45% to 52% [5,6,7,8,9,10]. Developing effective, low cost, and scalable interventions to effectively support adherence to diabetes medication is, therefore, urgently needed [11].

Health interventions that are delivered via mobile devices (often referred to as mHealth interventions) offer a new approach to support medication adherence [12]. Automated messages that are delivered via such devices can potentially target a wide range of beliefs and behaviours over a long period of time. The use of mobile devices permits the interventions to be wide-ranging and low cost and allows patients to choose the type of messages that they receive. The content of mHealth interventions can also be personalized based on data from electronic health records; and therefore, this type of intervention has great potential for delivering personalized health services. Although the available evidence suggests that mHealth interventions could improve the adherence to diabetes medication [12,13,14,15] and glycaemic control [14,15,16,17,18], in Spain this type of intervention is not available and remains largely under-researched.

In this context the Research Group in Prevention and Promotion of Health from the Balearic Islands in collaboration with the Public Health System of the Balearic Islands set out to design and develop DiabeText, a new intervention that is based on the use of a mobile-device system delivering automated, tailored, brief text messages (SMS type) to support medication adherence to antidiabetic drugs in people with T2DM. The Medical Research Council Guidelines for the development of complex interventions [19] underscore the need to take into account the perspectives of the relevant stakeholders as part of the design of complex interventions. The formative work exploring the views of primary care providers regarding the DiabeText intervention has been undertaken and will be available elsewhere. Formative qualitative research exploring the views of patients is key to gather end-user feedback concerning the intervention acceptability, the perceived utility [20], and to inform decisions about the intervention content and characteristics (frequency, timing, or tailoring, among others).

The aims of this qualitative study were threefold: to study the patients’ views on the acceptability and perceived utility of the proposed DiabeText intervention; to explore how the DiabeText intervention may address the unmet needs of people that are living with T2DM for medication-taking and healthy lifestyle behaviour; and to identify patient-elicited recommendations to optimize its potential impact in supporting adherence to diabetes medication. In the future, once DiabeText is fully developed and trialed, this mHealth tool will be offered to both public and private institutions for its potential implementation.

## 2. Materials and Methods

### 2.1. Participants

The inclusion criteria were adults (>18 years old) that were diagnosed with T2DM. Participant recruitment was assisted by primary care professionals, nurses that were working on diabetes education programs in primary care, and the regional diabetes charity (see acknowledgements). The sampling was purposive according to age group and educational level. This study was approved by the Research Ethics Committee of the Balearic Islands (CEI-IB) in July 2019 (39/48/19 PI).

### 2.2. Data Collection

We conducted four focus groups with 34 participants (62% men; mean (sd) age = 63 (9) years). The focus groups took place between October 2019 and January 2020. The participants were distributed into four groups (Table 1) according to their age (<65 vs. ≥65 years) and educational level (with university degree vs. with no university degree), as previous research shows that age and education level may be linked to differences in diabetes self-management (DSM), health literacy, and health-related expectations and needs [21,22,23,24,25].

The focus groups lasted approximately 60–75 min and were video-recorded to facilitate voice recognition of the participants if needed. Written consent was collected before each focus group began, and after the participant information sheet was handed out and questions were resolved. The focus groups were facilitated by members of the research team and took place in four primary health centres. To minimize the impact of the researchers on the data collection, all the researchers engaged in a reflexivity exercise, reflecting on their professional role and their personal assumptions about the intervention before the data collection commenced. A topic guide (Box 1) was developed prior to the commencement of the study.

Box 1 **Focus group guide that was developed ad hoc by the team based on objectives of the study and bibliography consultation.**

BARRIERS AND ENABLERS FOR T2DM SELF-MANAGEMENT

- We know that you all have diabetes. We wanted to start by asking: what do you do to care for and control sugar?
- What do you need to do to have sugar under control?
- What is more difficult for you to control?
- What are the things that help you? What works best for you?
- Regarding the recommendations on diet, what difficulties do you have in carrying out an adequate diet that helps you control your disease? What helps you overcome or face these difficulties?
- Regarding physical exercise, what difficulties do you have to carry out a level of physical exercise that helps you control your disease? What helps you overcome or face these difficulties?
- Regarding the diabetes medication, what do you think about diabetes medication? (Explore knowledge and beliefs on antidiabetic medication.) What difficulties do you have in taking the medication as prescribed by your doctor? What helps you overcome or face these difficulties?

ACCEPTABILITY AND PERCEIVED USEFULNESS OF THE PERSONALISED SMS TO HELP THEM CARRY OUT A BETTER DSM

- In general terms, what do you think of the idea of receiving SMS to your mobile phones with information that can help you take care of your diabetes?
- To what extent do you think it could be useful in your day-to-day life, as a tool to try to solve some of the problems you mentioned earlier?
- Do you have access to mobile phones?
- Do you see any problem accessing the text messages that we could send to your phones?

ASPECTS TO BE TAKEN INTO ACCOUNT TO MAXIMISE THE UTILITY AND ACCEPTABILITY OF THE PROPOSED SYSTEM

- In general, what aspects do you think we should take into account when starting this service, so that it has the best possible acceptance from patients?
- Would you prefer to receive the information in some other format other than SMS (audio, images or others)?
- What content do you think these messages should include?
- What are your preferences regarding:
  ◦ the language of the messages?
  ◦ the frequency of messages?
  ◦ the level of personalisation? (e.g., patient’s name)
  ◦ the ability for patients to customise the type of message that they want to receive? (content, frequency, language, time of day)
- Any other ideas or suggestions?

Following the established methods for qualitative research [26], the topic guide was used to steer the data collection process, rather than dictate it. During the focus groups, facilitators tried to ensure that everyone participated actively by insisting that there were no right or wrong answers, and that listening the points of view from all of the participants was key for the study. Co-facilitators (observers) took notes, paying particular attention to nonverbal communication. At the end of each focus group, the facilitator and the observers met for around 20 min to debrief. The debriefs focused on initial reflections on the data, exploration of first impressions emerging from the focus group, and the discussions about the main ideas that were proposed by the participants.

### 2.3. Data Analysis

The audio files were transcribed verbatim. The transcripts were read by the first author and crosschecked with the audiotapes or the videotapes to ensure the accuracy of the transcriptions. Before starting analysis, the research group shared their backgrounds and main preconceived ideas about the research to ensure their interests were not intruding on the data analysis process. A thematic approach that was based on Braun and Clark’s methodology [27,28] was used to analyse the data. The data were independently analysed by all members of research team (except AMB) and then discussed in a series of six meetings and at a workshop. All the data were coded by the lead author using an iterative approach. Initial notes were made, followed by a process of categorization and theme development. The initial themes on the acceptance and perceived utility of the short message service (SMS) system by patients were developed and later discussed with other members of the research team, who each analysed two transcripts by making notes, highlighting the issues of importance, and developing initial themes. Individual meetings with each researcher were held to discuss and progress the analysis. These culminated in a workshop with all the researchers to develop the final analysis and refine the themes. Discrepancies were solved by consensus. This approach allowed us to obtain greater interpretative and analytical wealth, and ensured the breadth of the data was incorporated in the analysis. All the researchers agreed on the final analysis and accepted it as being representative of the data.

We included in the results section a selected number of quotations (an extended, tabulated list of quotations is available in Table S1). This allowed us to ensure the voices of the participants are clearly present in the analysis, and that they served as a marker of rigor to demonstrate how our interpretation links to the data and helped to enrich our interpretation of the results.

## 3. Results

There were two main themes that emerged. The first theme encapsulated the patients’ perspectives on the unmet needs for DSM, and included three subthemes: (1) “lack of appropriate information is the key barrier for DSM”; (2) “support with medication is not perceived as a need, despite existing multiple barriers for medication-taking”; and (3) “support with diet and physical activity is perceived as important and demanded”. The second theme encapsulated the participants’ perceptions about DiabeText, a new text messaging intervention to support medication adherence, which included two subthemes: (1) “the intervention is acceptable and perceived as useful for DSM”, and (2) “specific characteristics that may enhance its usefulness”.

### 3.1. Patients’ Perspectives on Unmet Needs for DSM

#### 3.1.1. Lack of Appropriate Information Is the Key Barrier for DSM

The main barriers and enablers for DSM that were described by the participants are summarized in Table 2.

The lack of information about their condition and its causes emerged as a key barrier for DSM. The participants perceived their genetic background as the main trigger of their condition, even in the absence of a family history of T2DM. They had concerns about experiencing complications from T2DM, as they felt ill-informed about how to detect and manage early the warning signs, and about the burden that T2DM complications could pose in their lives.

For some, the information that was received during the consultations was perceived as overwhelming and difficult to understand and retain. Others cited the lack of time dedicated to consultations as prohibitive to gaining information. Equally, limited follow-up appointments were seen as contributing to a dearth of information.

*“What do I need to take? What type of insulin? What do I need to do? He [the primary care doctor] didn’t give me any damn kind of information”.*

*(Man, 59, without higher education.)*

Some participants, particularly those with higher educational attainment, reported they would like to receive more information and personalized training about T2DM. These patients seemed to feel responsible for controlling their illness and empowered to search, find, and take action to improve it. They expected clinical advice to be patient-centred, personalised, and updated.

*“You need to keep up to date with the advances in medication and with the techniques concerning that. If you don’t, the general practitioner gives you the same medication for ten years”.*

*(Man, 60, with higher education.)*

The participants perceived that the asymptomatic nature of T2DM was also a key barrier for its management. They referred to T2DM as the “silent disease”, because they felt it was difficult to notice when something goes wrong.

*“That’s the problem with diabetes, it doesn’t hurt, nothing happens (…) you don’t feel physical pain, and that’s the problem. You just get comfortable with it and then whoops!”*

*(Man, 53, with higher education.)*

#### 3.1.2. Support with Medication Is Not Perceived as a Need, despite Existing Multiple Barriers for Medication-Taking

In general, the participants felt they experience no major problems in adhering to their oral antidiabetic medication, and, therefore, suggested that they did not need support to take their pills as prescribed. However, some participants described difficulties taking their medication under specific circumstances, such as when eating out or travelling, whereas others struggled to manage their diabetes medication when having to take it in addition to a large number of pills each day.

*“I do carry my pill box, but there are far more things, like inhalers. There are just too many things and my memory just fails me…”.*

*(Man, <65 years old, without higher education.)*

Other patients experienced problems with getting their medication from the pharmacy, as it was only available during a specific period of time (in the Balearic Islands Health Service, chronic prescriptions can be dispensed up to ten days in advance but not earlier). However, some participants raised concerns about the need and usefulness of their diabetes medication, and a minority said they did not trust medication and questioned whether taking it could be detrimental for their health.

*“Some people are very reluctant to take the medication due to the amount, the contraindications and other problems. (…) they’ll only take their medication when there’s an emergency”.*

*(Woman, 66, with higher education.)*

*“I don’t believe that many pills are actually good”.*

*(Man, 60, with higher education.)*

#### 3.1.3. Support with Diet and Physical Activity Is Perceived as Important and Demanded

Following a healthy diet was perceived as a key aspect of DSM. Most participants were willing to engage in a healthy diet, but lacked the nutritional knowledge (sugar content of food, appropriate foods for their T2DM, or the recommended amount of starchy food and fruit among others), or cooking skills.

*“I go to the supermarkets and ask for the diabetic products, but no one knows anything to help me with my diet”.*

*(Woman, 74, without higher education.)*

One participant refused to engage in healthy eating because, while he acknowledged it may improve this specific condition, he felt it was not good for his overall wellbeing. He also felt that it may cause him stigma in his social life.

*“You may follow a strict diet (…) everything boiled, a chicken breast, etc., and you may cure your diabetes… But you will end up mad. You may end up with depression. (…) And then, well, you meet up with your friends and you can’t give up having a beer. So I don’t want to have any stigma. The pill and that’s it”.*

*(Man, 63, with higher education.)*

Although there was a general perception that physical activity is an important component of an adequate DSM and most participants reported an active lifestyle, some other participants felt they had no motivation to exercise as part of their everyday life. A lack of time, and of confidence doing physical activity emerged as significant barriers. The participants voiced doubts about being able to carry out physical activities that were appropriate to their individual needs and other co-existing health problems, indicating an unmet need for information about the impact of physical activity and DSM.

*“I have a question: Does it matter if you walk in the morning or in the afternoon? Is the point just to walk or is there a better time of the day? I ask because the strong meals usually happen during the day, so if you want to burn them, does it matter if you do so in the morning or in the evening?”*

*(Woman, 55, without higher education.)*

### 3.2. Participants’ Acceptability and Perceived Utility of the DiabeText Text Messaging Intervention

#### 3.2.1. The Proposed Messaging System Is Acceptable and Perceived as Useful for DSM

The majority of participants found DiabeText acceptable as they perceived that receiving information about T2DM via SMS could help them manage their condition. The participants felt that receiving frequent text messages could help them increase their awareness of their condition (frequently forgotten or neglected due to its asymptomatic nature) and remind them to take care of it.

In addition to receiving information about medical recommendations and upcoming follow-up appointments, patients also found it useful to receive additional information and training regarding medication, diet, physical exercise, and prevention of T2DM complications. They thought this would be helpful because they felt overwhelmed with the amount of information that they received during clinical appointments. They also perceived the intervention as a helpful strategy to support healthy lifestyle behaviours.

*“If I were told something about it, I would not eat sweets that day”.*

*(Woman, 72, without higher education.)*

The participants described other areas that the intervention should address, such symptoms of hypoglycaemia and upcoming foot and eye fundus check-up appointments.

A minority of the participants considered DiabeText as unnecessary for them because they perceived that they had no problems in managing their condition but acknowledged the potential usefulness for other patients who faced more difficulties managing their diabetes.

*“Helpful? No, not for me. Although it could be useful for older people, like for my mother. She needs to take her medication at 6 p.m., but if her alarm for the medication doesn’t go off at that time, I believe it could be 8 p.m. and she would have forgotten to take it”.*

*(Woman, 46, without higher education.)*

Delivering the intervention through a mobile phone was not perceived a barrier for most participants, who described themselves as regular smartphone users. The focus group of younger participants with university studies described how they used the Internet to obtain information about T2DM, as well as smartphone apps to help them manage their physical activity, body weight, or blood glucose levels.

*“I have a nice scale app that informs me about my weight, fat index, body mass… Because your diabetes decreases if you’re healthy. (…) I have that information in my phone so I can keep track of it.*

*(Man, 60, with higher education.)*

In the focus group of older participants with no university degree some participants felt they were not proficient at using their mobile phones, and others raised reading dexterity issues.

*“I don’t know how to use the phone. They gave me a list with the information I need if I need to call my daughter or my son. I just need to click where it says so and that’s it”.*

*(Woman, 75, without higher education.)*

#### 3.2.2. Specific Characteristics of the System That May Enhance the Usefulness of DiabeText

The patients discussed the characteristics that the intervention should include to be helpful for them. They talked about the language, tone, and comprehensibility of the messages, their frequency, the time during the day that was best to receive them, and other characteristics about format and personalisation (Table 3).

Participants suggested that the messages should be written in Spanish but also in Catalan (first language for some of them). They recommended that messages be clear and concise so they could be read quickly and easily understood.

*“Text messages should be simple and clear like a manual”.*

*(Man, 60, with higher education.)*

The participants underscored the need for a positive and motivating tone, so they feel encouraged and motivated to follow the messages’ recommendations. They differed in terms of their preference regarding the frequency and the time of the day, indicating that these characteristics may require a personalized approach. They also proposed personalized messages according to their physical activity level, or their cooking and Internet skills. They suggested other characteristics such as the use of images and links, and the possibility to reply to the messages to allow bidirectional communication.

*“I believe it would be a good idea if you could answer to that information. (…) I believe that would be a positive aspect”.*

*(Man, 53, with higher education.)*

## 4. Discussion

In this qualitative study with 34 patients with T2DM, we studied patients’ perceptions and experiences about DSM, and identified a large number of barriers for an adequate adherence to medication treatment and lifestyle recommendations. An intervention that was based on the use of short text messages to patients’ mobile devices was perceived as acceptable and as potentially useful. To meet the needs of this population group, the intervention should deliver DSM education focusing not only on supporting medication adherence but also lifestyle change behaviour, with the ultimate goals of raising awareness, improving knowledge, and increasing self-efficacy of DSM. Personalizing the content, language, and frequency of the messages, and allowing bidirectional communication, may further enhance patient experiences in using the messaging intervention.

Information about how to adequately self-manage T2DM consistently emerged as a key unmet need in the four focus groups. The overloaded schedule of primary health professionals, the short duration of consultations, and the lack of continuity of care are well known factors hindering the delivery of DSM education as part of routine clinical practice [29,30]. Adequate DSM requires specialized advice on diet, exercise, and prevention of complications, which should be delivered by coordinated teams that are working together. However, in our community the coordination between the primary and specialized teams still remains a challenge [31,32,33,34,35], and do not include some specialists such as dietitians-nutritionists [36], psychotherapists [37,38], or chiropodists [39].

Although some concerns were raised about the usefulness of their medication, the participants in our study generally perceived having no problems taking their oral diabetes medication as prescribed and suggested that new strategies to support lifestyle change behaviour were more urgently needed. This is in line with a recent qualitative study, which observed that T2DM patients felt more comfortable taking responsibility for medication than diet and exercise [40]. However, multiple studies show that in Spain around 50% of the patients do not adhere to their prescribed oral antidiabetic drugs [5,6,7,8,9,10]. As suggested by a recent meta-synthesis, patients may have no problems in understanding the need for medications and how to manage them, but they may deliberately choose to adjust the dosage and timing in their daily lives [41]. Data from our study suggested that the lack of symptoms and the perceived relationship between medication and diet could also influence adherence.

We observed that patients with a lower education level described more problems, reported less engagement with DSM, and more passive reliance on primary care professionals than those with higher education qualifications. This is consistent with the previous literature, which shows an association between a lower education level and lower DSM and self-efficacy [21,24,25,42]. Both education and knowledge degree are determinant factors of the attitudes of patients to take pills and follow a healthy lifestyle [30,43].

In terms of intervention acceptability, our findings are in line with previous studies which show that text messaging interventions present high patient acceptability. A recent qualitative study with 24 people with T2DM with a low educational level and living in a low-resource settings [44], observed that a text messaging intervention presented an adequate acceptability in terms of its actual use, frequency, and the role of texts as a reminder. In another study with 80 adults with T2DM [45], the user experience was favorable in all groups but mostly among younger patients and those that were more recently diagnosed with T2DM. In this study, people with a lower educational level were more prone to express negative views about the proposed intervention, and referred more digital literacy-related barriers to receive and read text messages in their phones. Social support (from friends and family) was perceived as key requisite to overcome these limitations. In a recent qualitative study with 21 participants with T2DM, they also described technical proficiency as a main barrier to access and use of information and communication technologies, but they were motivated to receive social support to use them because they provided self-care support, a feeling of control over T2D, and personalized advice or feedback [46]. In fact, some participants self-identified as early adopters and technophiles, while others felt less able to navigate new innovations but were still using them, as we also described in this study. However, digital literacy among the general population in Spain depends on sociodemographic factors (sex, age, education, and income level) which should be taken into account to design new mHealth tools to avoid health inequalities [47].

A key driver of acceptability is the perceived relevance of the intervention [48]. If messages are not perceived as relevant, people with T2DM may refuse to use it. In that sense, those participants who felt more confident with the management of their condition perceived that the proposed intervention was unnecessary for themselves, but acknowledged the potential usefulness for other patients. Tailoring the intervention according to participants preferences and needs is, therefore, a key aspect not only for optimizing the impact of the intervention, but also to ensure it is accepted and used by a wide range of patients. Patients identified a series of topics such as: medication-taking reminders and check-ups for the diabetic foot and eye fundus, motivational messages for doing exercise, informative messages about T2DM and its complications, and nutritional messages. Similarly, in a study with 63 South Asian people with T2DM that were living in the UK, their preferences for message content were about medication-related information, information about diet and natural approaches, information about physical activity, and other information such as stress management, new research findings, details of local diabetes-related events, or “reversing diabetes” [49]. Another key characteristic that text messages should have, based in patients’ preferences, is that they remain motivational. As in a recent study, the authors reported that patients preferred simple and positively-framed communication [50]. Receiving novel information about diabetes medications, emotional support, and reminders to take medication were identified as the most helpful aspects of the intervention.

Recent research [51] shows that it is technologically feasible to personalize a text messaging intervention based on data from electronic health reports (e.g., medication dispensation, presence of comorbidities, tests results), from patient-reported behaviours (using validated questionnaires to measure adherence to medication and diet and physical activity-related behaviours), and from patient-reported preferences about the intervention (frequency of the messages, timing, topic of especial interest the messages should address). In this sense, text messages can fit the preferences of patients in terms of their content, but they can also be tailored depending on personalized clinical data.

### Strengths and Limitations

To our knowledge, this is the first study that was carried out in Spain that assesses the patients’ perspectives and preferences about a tailored text message intervention to improve medication adherence. An important strength of this study is its methodological rigor. The study meets the main trustworthiness criteria: credibility, dependability, transferability, and confirmability [52]. The analysis categories comply with the criteria of comprehensiveness, relevancy, and objectivity. Our study has also some limitations. First, the participants were a relatively homogeneous group in terms of ethnicity (white) and nationality (Spanish). Therefore, we cannot extrapolate these results to other groups. Second, we cannot rule out a potential selection (participation) bias, as those patients agreeing to take part in our focus group study may be more activated toward the management of their condition than those who chose not to participate. Similarly, the fact that patients were recruited through peer-led T2DM educational programs and through the local diabetes charity may have reduced the representativeness of our sample in relation to the general population of people with T2DM in Majorca. Also, in some cases, some focus group participants may find it difficult to express their views. Efforts were made to include all participants and elicit their views. Finally, it is worth noting that this study was previous to the COVID-19 pandemic. The views and perspectives of the patients may have changed as a result of the reorganization of health services that have been imposed since then, where telematics consultations are the norm rather than the exception.

## 5. Conclusions

The proposed DiabeText intervention is well accepted by patients who reported unmet needs in terms of DSM education to support not only medication-taking, but more prominently lifestyle change behaviour. Since unmet needs are likely to differ according to patients’ educational level and age, tailoring the intervention according to their preferences and needs is a key aspect to ensure the intervention is perceived as relevant and it is used by a wide range of patient profiles. Access to, and system integration with, electronic health records is a necessary requisite for the implementation of such personalization features.

## Figures and Tables

**Table 1:** Focus groups composition.

Groups	Number of Participants	Gender Distribution (Male/Female)	Age Range (Years)	Focus Group Duration (Minutes)
Aged < 65; without higher education	10	6/4	46–64	63
Aged ≥ 65; without higher education	8	5/3	68–74	66
Aged < 65; with higher education	9	6/3	43–63	73
Aged ≥ 65; with higher education	7	4/3	66–75	55

**Table 2:** Barriers and enablers for DSM that were identified from four focus group discussions with people with type 2 diabetes in the Balearic Islands (Spain) during October 2019 and January 2020.

	Barriers	Enablers
Overall management	Stigmatisation Asymptomatic nature of the condition Receiving too much information at the same time Not receiving enough information because of the lack of time during consultations or poor follow-up	Having a good relationship with the doctor and the nurse Blood glucose self-monitoring Support from friends and family Support from peers
Medication	Forgetting the medication Fear of side effects Management of polypharmacy Difficulties taking medication away from home or traveling Misunderstanding of medical advice Trouble with dispensation from the pharmacy	Going to medical appointments Putting alarms Using a pillbox Trusting the medical doctor Receiving information on how the medication works
Diet	Fear of being scolded for not complying with proposed dietary guidelines Lack of specialised advice, counselling and support on nutrition and dietetics Misinformation on the Internet Eating out	Cooking skills New recipes ideas Body weight follow-up Motivational skills
Physical activity	Long work shifts and lack of leisure time Lack of specialised advice, counselling and support on sports Physical problems	Awareness of the benefits of physical exercise to control blood glucose Feeling confident with practising sports Walking with others Counting steps
T2DM complications	Lack of time and communication with the healthcare professional Too many appointments	Being informed about T2DM complications and their management Nursing follow-up

**Table 3:** Characteristics of the messages that were suggested by participants from four focus groups with people with type 2 diabetes in the Balearic Islands (Spain) during October 2019 and January 2020.

Characteristics of Messages	Participants’ Opinion
Language	Spanish Catalan
Comprehension	Clear Short
Tone	Positive Motivating
Frequency	Every day Three to five times per week Once a month
Time of day	Early in the morning Avoid working hours Not at night
Level of personalisation	Physical activity level Frequency of messages Recipes Links for people with Internet on the mobile phone
Other characteristics	Giving and receiving feedback through the system would be valuable Images would help to get the information in a better way

## Supplementary Materials

The following supporting information can be downloaded at: https://www.mdpi.com/article/10.3390/ijerph19031902/s1, Table S1: Extended list of quotations from participants associated to each theme and subthemes identified.
