# Supplementary material for: Patients’ Views on the Design of DiabeText, a New mHealth Intervention to Improve Adherence to Oral Antidiabetes Medication in Spain: A Qualitative Study
Source: Int J Environ Res Public Health. 2022 Feb 8;19(3):1902. doi: 10.3390/ijerph19031902 (PMC8834698; doi:10.3390/ijerph19031902)
Supplement: Supplementary file 1 [file ijerph-19-01902-s001.zip › ijerph-1545309-supplementary.pdf]

Table S1. Extended list of quotations from participants associated to each theme and subthemes identified.

Theme 1. Patients' perspectives on unmet needs for DSM	
Subthemes	Quotations
Lack of appropriate information is the key barrier for DSM	"We have a serious problem. Most of us have a computer, so we look for information on diabetes. This information misinforms us. It holds us back because we are not doctors, we don't have their mentality or their studies, so we understand it differently depending on what we want. Sure, the computer is there to inform us, but it surely does misinform us too". Man, 64, without higher education.<br><br>"Well, society is also responsible. They label you; if you are diabetic then they regard you as 'you poor thing'. I wouldn't like to be referred to as a 'poor thing', I am a normal person. I have my problems, as everyone else, but it may also be the distrust people with diabetes have regarding unfamiliar surroundings. As for the familiar ones, I do recommend they know you are diabetic". Man, 53, with higher education.<br><br>"It's good to be reminded. Many times, you get comfortable with it or feel good so you ignore it… That's the problem with diabetes, it doesn't hurt, nothing happens… Well, yeah, if you don't pay certain attention, you may get a headache or something in the end, but you don't feel physical pain, and that's the problem. You just get comfortable with it and then whoops! So, I believe a reminder of the guidelines would be beneficial". Man, 53, with higher education.<br><br>"How many types of diabetes are there? An endocrinologist told me there are three: type 1, type 2 and diabetes mellitus. But I don't have type 1 diabetes, nor type 2 diabetes nor diabetes mellitus. I have the worst of them, but no one told me what kind that is". Man, 59, without higher education.<br><br>"It has been very difficult for me to assume that I have diabetes, even though there are diabetics in my family, and, of course, when you do not assume it, you do not obey. You think nothing is that serious. A cookie. Nothing. One ice cream a week does nothing. But everything does." Woman, 70, with higher education.<br><br>"I'd like to know that there are many people who know when there is a rise as well as a fall. Honestly, I never notice". Man, < 65 years old (unknown age), without higher education.<br><br>"I have come across many people who say: "I have a little sugar." No, darling, it doesn't go that way, you don't have a little sugar, you have diabetes, and since you don't control it, when you're 70 years old, I don't know what you're going to have." Woman, 61, with higher education.<br><br>"I think, in my case, that, maybe, I put on a bandage, and I lack that awareness that she has, so constant, of facing this, of being aware that, indeed, you have to take control of this. And, to put it in some way, you sail aimlessly every day, saying: "well, we'll see what happens." Man, 60, with higher education.<br><br>"Look, I think my doctor is a wonderful man. He makes me sit down, shakes my hand and says: 'Hello [name], how are you?'. So, I answer: 'I'm very well'. If they hadn't told I'm diabetic I would believe I don't have anything. The silent disease indeed. He laughs, and I tell him my things. He looks at me with an angelical face; he is really beautiful on the inside. He even looks like a priest". Woman, 70, with higher education.<br><br>"I got mad at him [his doctor] and I didn't come back for five years, and I told him so: 'I'm not coming back because you don't give me any kind of information'. His glasses sliding down, the medical test there, and that's it, goodbye and see you in six months. What's that? But hey, I just underwent surgery. I have undergone surgery four times and you are not helping at all. You don't tell me which pills I need to take or what type of insulin… You don't say anything. So, I

Theme 1. Patients' perspectives on unmet needs for DSM	
Subthemes	Quotations
	reported that to the chief health inspector, who told me I still needed to go visit him. I told him that I wasn't saying I didn't need to go, but that I needed information. What do I need to take? What type of insulin? What do I need to do? Yet he didn't give me any damn kind of information". Man, 59, without higher education.<br><br>"The first problem is found at the health centre because they don't make a customised diet plan for you. They have a standard one that they give you and that's it. The physician doesn't prescribe for you either. They give you a standard treatment and that's it. And if you don't come back later, nobody calls you. You are on your own. That's the problem: there's no efficient prevention system at the health centre; or at least it wasn't last time I went, which was a long time ago. I have to go to a private centre because they do attend to me there. I go to two different ones. But if I didn't, the endocrinologist wouldn't attend to me because you need to be a wreck to be attended by the endocrinologist. So, it is deficient and you need to look out for yourself". Man, 60, with higher education.<br><br>"You need to keep up to date with the advances in medication and with the techniques concerning that. If you don't, the general practitioner gives you the same medication for ten years. So, by keeping up to date with that, you can insist a bit and claim some medication is out and ask about their opinion on that. Otherwise, I have to look it up on the Internet, which occasionally I do, but the other option would be interesting. Because the GP is not up to date sometimes". Man, 60, with higher education.<br><br>"And to personalize based on the medical history, wouldn't it be interesting, perhaps, when you debut, if there was a reference to your personality? That is, if you do a triathlon, you will not send it to walk. But for me, for example, don't send me to do triathlon, because I'm going to send you to hell. That is, take into account the personality of each one: can I walk? Yes, I can walk. And as for food the same." Woman, 61, with higher education.<br><br>"Have the doctor tell you: "Buy yourself a dog, take it, for a walk." There is a part of listening to you about how your life is and that someone detects that you are a hot dog, that you are a sedentary person, and how you can change it. And, apart, that the changes are little by little and with habits, no: "you have to do so many kilometers". No. Change habits, and healthy habits." Man, 60, with higher education.<br><br>"Breathe through the pine forest, see that. It is that perhaps one needs to feel that loneliness." Man, 60, with higher education.<br><br>"Knowing what the pill you are taking does, will probably help you understand and be more aware of what happens if you take that pill or not". Man, 56, with higher education.<br><br>"I have a question. Isn't diabetes a chronic disease? Incurable? How this man says that diabetes can be cured. It is a question that I have. Besides, normally my life is a normal life. I have had a heart transplant, and the truth is that I am a very active person, I follow a regimen, but not a strict regimen either. I am not a vegetable man, if I tell you the truth. I eat a lot of grilled chicken, turkey, but I don't usually eat vegetables to take care of myself, I don't. I eat a little of everything. Sometimes I spend a little on something, because temptation can. But in the sugar, I don't know if it is a weak sugar or I don't know what. I have it under control. I have never had big ups or lows. I prick myself every two or three weeks. I am very controlled by the analysis that they do to me." Man, unknown age, without higher education.
Support with medication is not perceived as a	"I know what I need to do regarding my medication every day, so I don't need it. My sugar level dropped four times, until reaching 30, but no one needs to remind me what I need to take". Man, unknown age, without higher education.

Theme 1. Patients' perspectives on unmet needs for DSM	
Subthemes	Quotations
need, despite existing multiple barriers for medication taking	"As for the pills, I do not need a reminder for them. I always keep them in mind. As for recipes and diets, which are important too, I have problems dealing with those". Woman, 74, without higher education.<br><br>"I take pills for the heart and for the COPD because I also have COPD… So, it's impossible". Man, unknown age, without higher education.<br><br>"If you are in the city and then you first go to [place name]and then to [place name], you cannot carry the pills. Well, I do carry my pill box, but there are far more things, like inhalers. There are just too many things and my memory just fails me…". Man, unknown age, without higher education.<br><br>"If they give you the pills for a month and you take 30. But some months have 31 days. Because one day don't take the pill, nothing happens." Man, 68, without higher education.<br><br>"Yes, I sometimes talk to the girls at the pharmacy, and they tell me: "there are people who picked up the pills two days ago", because, of course, everything is recorded on the card, "and they come and say they need the same pills ". It says: "They came two days ago, where have they put them?"" Woman, 71, without higher education.<br><br>"Some people are very reluctant to the medication due to the amount, the contraindications and other problems. They are reluctant in the sense that they'll only take their medication when there's an emergency". Woman, 66, with higher education.<br><br>"I don't believe that many pills are actually good". Man, 60, with higher education.
Support with diet and physical activity is perceived as important and demanded	"I follow the medication instructions with no problem, but as for the diet… I can't say the same. I believe I don't follow it, although occasionally I intend to and I fret over it. Yet if I see it on the phone, I say to myself that I can't do it. The diet would be very beneficial to me". Man, 68, without higher education.<br><br>"Well, I'm diabetic but not insulin dependent. Metformin is my treatment, and for about half a year I had quite high levels; high meaning 150-160. The reason is I lacked one of the three factors that you mentioned: the diet". Man, 75, with higher education.<br><br>"I spoke to a friend of mine who is a chemist. He told me it had no sugar, so I asked him why my sugar level increases then… Because of the carbohydrates he said". Man, 59, without higher education.<br><br>"I go to the supermarkets and ask for the diabetic products, but no one answers". Woman, 74, without higher education.<br><br>"I have looked for and downloaded lots of information on the Internet. For example, he was talking about eating a lot of bread, but don't forget about pasta, potatoes, etc. I mean the carbohydrates. Maybe there is a lack of information on the quantities one can take". Woman, 66, with higher education.<br><br>"You may follow a strict diet of those, those with whatever foodstuffs, with everything boiled, a chicken breast, etc., and you may cure your diabetes… But you will end up mad. You may end up with a depression". Man, 63, with higher education.

Theme 1. Patients' perspectives on unmet needs for DSM	
Subthemes	Quotations
	"In my case, apart from the medication my doctor prescribes me, the diet. I started cycling, but walking… I don't walk. For one reason or another, I don't walk, so I ride a stationary bike". Woman, 54, without higher education.<br><br>"I gifted myself a stationary bike for Mother's Day. I ride for half an hour, three quarters, an hour… It doesn't matter if its 10:00 p.m., if I'm watching the TV, or if it's the weekend and I'm there to clean the house. Is it comparable to walking half an hour?" Woman, 54, without higher education.<br><br>"I asked a question: how much are 10,000 steps? Sometimes I walked for an hour thinking I did 10,000 steps, but it turns out I need to walk two times that if I want to do 10,000 steps". Man, 60, with higher education.<br><br>"I have a question: Does it matter if you walk in the morning or in the afternoon? Is the point just to walk or is there a better time of the day? I ask because the strong meals usually happen during the day, so if you want to burn them, does it matter if you do so in the morning or in the evening?" Woman, 55, without higher education.

Theme 2. Participants’ acceptability and perceived utility of DiabeText	
Subthemes	Quotations
The proposed messaging system is acceptable and perceived as useful for DSM	“It’s good to be reminded. Many times you get comfortable with it or feel good so you ignore it… That’s the problem with diabetes, it doesn’t hurt, nothing happens… Well, yeah, if you don’t pay certain attention to it you may get a headache or something in the end, but you don’t feel physical pain, and that’s the problem. You just get comfortable with it and then whoops! So I believe a reminder of the guidelines would be beneficial”. Man, 53, with higher education.<br><br>“Always keeping an eye on the disease is good. We shouldn’t forget it. This disease is not a bother, so I believe that is the reason why some people don’t take pills. If it bothered, if it hurt, I’m sure we all would take the pills. But it seems the disease is nowhere to be found, you feel good, so you often forget about it”. Man, 75, with higher education.<br><br>“The text messages could be about hypoglycaemia, which is really bad. I never had one yet now I’ve already had two. Now I’m more careful, so I do notice. Sometimes I forget to eat. They say it’s five meals a day, but sometimes I forget”. Woman, 70, with higher education.<br><br>“I think that if I were told something about it, I would not eat sweets that day”. Woman, 72, without higher education.<br><br>“My endocrinologist instructs me once a year to do that thing related to the diabetic eye disease and the diabetic foot. That could also be interesting because there might be people who never do that”. Woman, 66, with higher education.<br><br>“Everything is useful, but it depends on how you use it. I mean, a reminder of yours telling me to take the pill is useful because it reminds me. But it depends on what I do with that. I mean, if I don’t have the pills nearby at that moment, I say to myself that I’ll take them later. Yet when later comes, I’ve forgotten already. The device can tell you that you need to follow the diet, but you might answer with just a ‘yes, I know, thanks’ and that’s it”. Man, 64, without higher education.<br><br>“You need to be determined about it. Even if the alarm goes off for you to start walking you may just dismiss it and that’s it. It depends on the person if they will comply or not. Everyone should know what they need to do, I believe we all do. Even if you don’t, you do know… No, I wouldn’t need that message”. Man, < 65 years old (unknown age), without higher education.<br><br>“Helpful? No, not for me. Although it could be useful for older people, like for my mother. She needs to take her medication at 6 p.m., but if her alarm for the medication doesn’t go off at that time, I believe it could be 8 p.m. and she would already have forgotten to take it. Maybe it’s not useful for us, but maybe it is for other people”. Woman, 46, without higher education.<br><br>“I have it under control thanks to the mobile phone. I take at least 25 pills, which I take at six or seven different times: some at 7 a.m., another set at 8 a.m., some others at 2 p.m., and at 7 p.m., and at 10 p.m. and at 12 a.m. There are too many, so you could easily forget about one. That’s the reason why I have an alarm for everything”. Man, < 65 years old (unknown age), without higher education.<br><br>“I can tell you what I have in my phone: alarms, mainly. Like for the fundus of the eye, I can’t forget about that one. It just happens once or twice a year. Also, for the time of the medication. I have one alarm set at noon, but none in the morning or at night. I also have a nice scale app that informs me about my weight, fat index, body mass… Because your diabetes decreases if you’re healthy. I mean, you need to be on your weight, on your body mass and all that. I have that

Theme 2. Participants' acceptability and perceived utility of DiabeText	
Subthemes	Quotations
	information also in my phone so I can keep track of it. You need to weigh yourself". Man, 60, with higher education.<br><br>"I don't know how to use the phone. They gave me a list with the information I need if I need to call my daughter or my son. I just need to click where it says so and that's it". Woman, 75, without higher education.<br><br>"I can't read much, but I read it. That's why I tell you that I might need three times to read it, but I do read it". Man, 68, without higher education.
Specific characteristics of the system that may determine the usefulness of DiabeText	"Text messages should be simple and clear like a manual". Man, 60, with higher education.<br><br>"I'd prefer them to be in a positive language. They should never say things like 'you can't do this' or 'don't do that'. Absolutely not". Woman, 61, with higher education.<br><br>"Maybe there are people who don't need text messages for everything. Some people may have problems with the diet and I, for example, have problems with doing exercise. I'd rather receive messages about different strategies for exercising". Woman, 61, with higher education.<br><br>"I believe it would be a good idea if you could answer to that information. It doesn't need to be over the phone. There should be some channel of communication, like an email, so you can respond in relation to the last text message, for example. I believe that would be a positive aspect". Man, 53, with higher education.
